# Supplementary material for: Metabolomic Markers for the Early Selection of Coffea canephora Plants with Desirable Cup Quality Traits
Source: Metabolites. 2019 Oct 4;9(10):214. doi: 10.3390/metabo9100214 (PMC6835713; doi:10.3390/metabo9100214)
Supplement: Supplementary file 1 [file metabolites-09-00214-s001.pdf]

metabolites

Metabolomic Markers for the Early Selection of *Coffea canephora* Plants with Desirable Cup Quality Traits

Roberto Gamboa-Becerra [1,2,*], María Cecilia Hernández-Hernández [3], Óscar González-Ríos [3], Mirna L. Suárez-Quiroz [3], Eligio Gálvez-Ponce [4], José Juan Ordaz-Ortiz [5] and Robert Winkler [1,*]

Supplementary Material

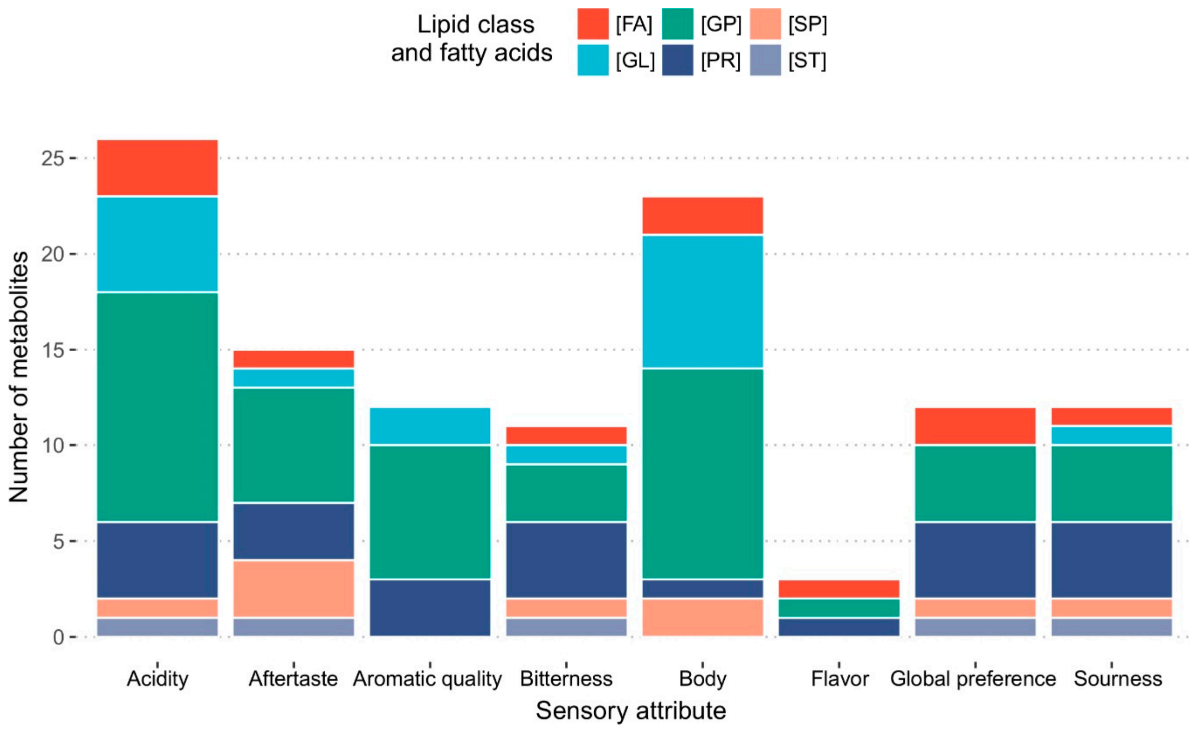

Figure S1. Lipid classes of coffee roasted beans linked to sensory traits. [FA]: Fatty acids; [GL]: Glycerolipids; [GP]: Glycerophospholipids; [PR]: Prenol lipids; [SP]: Sphingolipids; [ST]: sterol lipids.

Table S1. Metabolites of roasted coffee beans linked to coffee cup quality.

Sensory attribute	Metabolite (*m/z*)	Compound class	*p*-Value	SCC
Acidity	PC(18:3(6Z,9Z,12Z)/18:1(9Z))	Lipids [GP]	7.16 x 10^{-6}	0.814
Acidity	Trigonelline	Alkaloids	1.35 x 10^{-4}	0.738
Acidity	Neoxanthin	Carotenoids	3.37 x 10^{-4}	0.707
Acidity	Astaxanthin diglucoside	Lipids [PR]	4.64 x 10^{-4}	0.696
Acidity	3-keto-4-methylzymosterol	Lipids [ST]	5.52 x 10^{-4}	0.689
Acidity	5,9-tetracosadienoic acid	Fatty acids	5.80 x 10^{-4}	0.687
Acidity	PA(12:0/12:0)	Lipids [GP]	6.42 x 10^{-4}	0.683
Acidity	Tricetin 7,3',4',5'-trimethyl eter 5-xylosyl-(1->2)-rhamnoside	Flavonoids	6.86 x 10^{-4}	0.681
Acidity	m/z 498.7039	-	9.46 x 10^{-4}	0.668
Acidity	m/z 520.8069	-	1.45 x 10^{-3}	0.649
Acidity	m/z 843.6908	-	1.98 x 10^{-3}	0.635
Acidity	m/z 727.9535	-	2.25 x 10^{-3}	0.629
Acidity	m/z 963.4788	-	2.64 x 10^{-3}	0.621
Acidity	PC(16:0/18:1(9Z))	Lipids [GP]	3.18 x 10^{-3}	0.612
Acidity	m/z 729.2108	-	3.71 x 10^{-3}	0.604
Acidity	1-Palmitoylglycerophosphocholine	Lipids [GP]	4.00 x 10^{-3}	0.601
Acidity	PG(O-20:0/17:2(9Z,12Z))	Lipids [GP]	4.92 x 10^{-3}	0.589
Acidity	m/z 498.8341	-	5.29 x 10^{-3}	0.586
Acidity	Pelargonidin-3,5-diglucoside	Flavonoids	5.55 x 10^{-3}	0.583
Acidity	TG(16:1/18:2/18:2)[iso3]	Lipids [GL]	6.16 x 10^{-3}	0.577
Acidity	TG(20:2(11Z,14Z)	Lipids [GL]	7.15 x 10^{-3}	0.569
Acidity	m/z 743.6496	-	7.39 x 10^{-3}	0.567
Acidity	PC(24:0/P-18:1(11Z))	Lipids [GP]	7.91 x 10^{-3}	0.563
Acidity	3-Galloylprocyanidin B1	Flavonoids	8.63 x 10^{-3}	0.558
Acidity	PC(18:1(11Z)/18:1(9Z))	Lipids [GP]	8.63 x 10^{-3}	0.558
Acidity	Quercetin 4′-glucoside	Flavonoids	8.92 x 10^{-3}	0.556
Acidity	m/z 576.0403	-	9.31 x 10^{-3}	0.553
Acidity	m/z 715.4369	-	1.07 x 10^{-2}	0.545
Acidity	m/z 780.8105	-	1.08 x 10^{-2}	0.544
Acidity	m/z 818.6563	-	1.27 x 10^{-2}	0.534
Acidity	Gamma-aminobutyric acid	Organic acids	1.33 x 10^{-2}	0.531
Acidity	PC(17:2(9Z,12Z)/17:0)	Lipids [GP]	1.37 x 10^{-2}	0.529
Acidity	hydroxymethylglutaryl-CoA	Fatty acids	1.37 x 10^{-2}	0.529
Acidity	TG(18:2_18:2_19:1)	Lipids [GL]	1.38 x 10^{-2}	0.528
Acidity	m/z 469.9502	-	1.41 x 10^{-2}	0.527
Acidity	PG(18:0/18:3(6Z,9Z,12Z))	Lipids [GP]	1.82 x 10^{-2}	0.51
Acidity	PC(18:1(11Z)/18:2(9Z,12Z))	Lipids [GP]	1.85 x 10^{-2}	0.509
Acidity	m/z 816.64	-	2.05 x 10^{-2}	0.502
Acidity	m/z 886.9232	-	1.13 x 10^{-6}	-0.849
Acidity	m/z 665.7307	-	1.29 x 10^{-5}	-0.801
Acidity	Man3GlcNAcFucGlcNAc	Carbohydrates	1.90 x 10^{-5}	-0.792
Acidity	m/z 854.7416	-	3.80 x 10^{-5}	-0.774
Acidity	PS(12:0/15:0)	Lipids [GP]	8.41 x 10^{-5}	-0.752

Acidity	m/z 713.2618	-	1.46×10^{-4}	-0.735
Acidity	Undecaprenyl diphosphate	Lipids [PR]	2.85×10^{-4}	-0.713
Acidity	m/z 667.0015	-	8.74×10^{-4}	-0.671
Acidity	PS(20:2(11Z,14Z)/22:1(11Z))	Lipids [GP]	9.91×10^{-4}	-0.666
Acidity	Phytofluene	Carotenoids	1.27×10^{-3}	-0.655
Acidity	m/z 914.9457	-	1.32×10^{-3}	-0.653
Acidity	Glutaryl-CoA	Fatty acids	1.36×10^{-3}	-0.652
Acidity	Undecaprenyl diphosphate	Lipids [PR]	1.75×10^{-3}	-0.641
Acidity	m/z 690.8389	-	1.88×10^{-3}	-0.638
Acidity	UDP-3-O-(3-hydroxymyristoyl)-N-acetylglucosamine)	Carbohydrates	1.90×10^{-3}	-0.637
Acidity	m/z 884.9105	-	2.10×10^{-3}	-0.632
Acidity	m/z 857.0291	-	2.31×10^{-3}	-0.628
Acidity	m/z 659.7348	-	3.61×10^{-3}	-0.606
Acidity	m/z 552.6861	-	3.90×10^{-3}	-0.602
Acidity	a-Zeacarotene	Carotenoids	4.15×10^{-3}	-0.599
Acidity	m/z 581.1178	-	4.15×10^{-3}	-0.599
Acidity	Niazidin	Carbohydrates	5.04×10^{-3}	-0.588
Acidity	Apigenin 6-C-arabinoside 8-C-glucoside	Flavonoids	5.04×10^{-3}	-0.588
Acidity	LacCer(d18:0/22:0)	Lipids [SP]	5.10×10^{-3}	-0.588
Acidity	TG(18:2/18:3/20:0)[iso6]	Lipids [GL]	5.48×10^{-3}	-0.584
Acidity	Quercetin 3-(2''-(E)-caffeoyl-alpha-L-arabinopyranosyl)-(1->6)-glucoside	Flavonoids	5.55×10^{-3}	-0.583
Acidity	m/z 740.3981	-	5.74×10^{-3}	-0.581
Acidity	Nicotinic acid	Alkaloids	6.60×10^{-3}	-0.573
Acidity	m/z 821.605	-	7.91×10^{-3}	-0.563
Acidity	m/z 573.9349	-	8.82×10^{-3}	-0.556
Acidity	All-trans-dodecaprenyl diphosphate	Lipids [PR]	1.13×10^{-2}	-0.541
Acidity	m/z 693.5954	-	1.13×10^{-2}	-0.541
Acidity	m/z 850.5936	-	1.47×10^{-2}	-0.524
Acidity	Pelargonidin 3-(6''-succinyl-glucoside)	Flavonoids	1.56×10^{-2}	-0.52
Acidity	m/z 822.8373	-	1.63×10^{-2}	-0.517
Acidity	TG(14:0/20:5/20:5)	Lipids [GL]	1.76×10^{-2}	-0.512
Aftertaste	m/z 713.2618	-	8.18×10^{-5}	0.753
Aftertaste	m/z 886.9232	-	3.24×10^{-4}	0.709
Aftertaste	m/z 884.9105	-	1.02×10^{-3}	0.664
Aftertaste	3,5-dicaffeoylquinic acid	Chlorogenic acids	1.10×10^{-3}	0.661
Aftertaste	DGTS(23:6/15:3)	Lipids [GP]	1.45×10^{-3}	0.649
Aftertaste	Pelargonidin 3-(6''-succinyl-glucoside)	Flavonoids	1.72×10^{-3}	0.641
Aftertaste	m/z 469.9502	-	3.10×10^{-3}	0.614
Aftertaste	Glutaryl-CoA	Fatty acids	3.86×10^{-3}	0.602
Aftertaste	m/z 737.1965	-	4.06×10^{-3}	0.6
Aftertaste	All-trans-dodecaprenyl diphosphate	Lipids [PR]	4.70×10^{-3}	0.592
Aftertaste	Hederagenin	Terpenoids	6.09×10^{-3}	0.578
Aftertaste	m/z 665.7307	-	6.18×10^{-3}	0.577
Aftertaste	Calcium (+)-pantothenate	Organic acids	6.85×10^{-3}	0.571
Aftertaste	Undecaprenyl diphosphate	Lipids [PR]	7.67×10^{-3}	0.565
Aftertaste	LacCer(d18:0/22:0)	Lipids [SP]	8.86×10^{-3}	0.556
Aftertaste	Man3GlcNAcFucGlcNAc	Carbohydrates	9.45×10^{-3}	0.552

Aftertaste	Glucoscilliroside	Lipids [ST]	9.45×10^{-3}	0.552
Aftertaste	4-Aminobenzoic acid	Organic acids	9.66×10^{-3}	0.551
Aftertaste	m/z 581.1178	-	1.06×10^{-2}	0.545
Aftertaste	m/z 973.826	-	1.09×10^{-2}	0.543
Aftertaste	1,26-Dicaffeoylhexacosanediol	Chlorogenic acids	1.18×10^{-2}	0.538
Aftertaste	Apigenin 6-C-arabinoside 8-C-glucoside	Flavonoids	1.58×10^{-2}	0.52
Aftertaste	m/z 821.605	-	1.59×10^{-2}	0.519
Aftertaste	m/z 733.5719	-	1.67×10^{-2}	0.516
Aftertaste	m/z 677.7246	-	1.73×10^{-2}	0.513
Aftertaste	m/z 653.6050	-	1.76×10^{-2}	0.512
Aftertaste	PE(18:4(6Z,9Z,12Z,15Z)/P-16:0)	Lipids [GP]	1.79×10^{-2}	0.511
Aftertaste	m/z 693.5954	-	1.97×10^{-2}	0.505
Aftertaste	m/z 902.9107	-	2.11×10^{-2}	0.5
Aftertaste	Citric acid	Organic acids	1.02×10^{-4}	-0.746
Aftertaste	Quercetin 4´-glucoside	Flavonoids	1.26×10^{-4}	-0.74
Aftertaste	m/z 727.9535	-	5.14×10^{-4}	-0.692
Aftertaste	m/z 963.4788	-	5.23×10^{-4}	-0.691
Aftertaste	DGTS(16:0/26:5)	Lipids [GP]	1.25×10^{-3}	-0.656
Aftertaste	PC(16:0/18:2(9Z,12Z))	Lipids [GP]	1.30×10^{-3}	-0.654
Aftertaste	Trigonelline	Alkaloids	1.36×10^{-3}	-0.652
Aftertaste	Cer(d14:2(4E,6E)/22:1(13Z)(2OH))	Lipids [SP]	2.31×10^{-3}	-0.628
Aftertaste	Cer(d18:0/26:0)	Lipids [SP]	4.64×10^{-3}	-0.593
Aftertaste	m/z 729.2108	-	6.93×10^{-3}	-0.57
Aftertaste	3-Galloylprocyanidin B1	Flavonoids	7.76×10^{-3}	-0.564
Aftertaste	Astaxanthin diglucoside	Lipids [PR]	7.76×10^{-3}	-0.564
Aftertaste	m/z 971.5906	-	1.14×10^{-2}	-0.54
Aftertaste	Neoxanthin	Carotenoids	1.33×10^{-2}	-0.531
Aftertaste	m/z 743.6496	-	1.32×10^{-2}	-0.531
Aftertaste	PA(12:0/12:0)	Lipids [GP]	1.53×10^{-2}	-0.522
Aftertaste	m/z 876.5869	-	1.63×10^{-2}	-0.517
Aftertaste	PC(18:3(6Z,9Z,12Z)/18:1(9Z))	Lipids [GP]	1.66×10^{-2}	-0.516
Aftertaste	DGTS(10:0/10:1)	Lipids [GL]	1.69×10^{-2}	-0.515
Aftertaste	m/z 674.8121	-	1.69×10^{-2}	-0.515
Aftertaste	m/z 722.7304	-	1.81×10^{-2}	-0.51
Aftertaste	m/z 868.6639	-	2.05×10^{-2}	-0.502
Aromatic quality	m/z 665.7307	-	4.08×10^{-4}	0.7
Aromatic quality	UDP-D-xylose	Carbohydrates	1.74×10^{-3}	0.641
Aromatic quality	m/z 737.1965	-	2.63×10^{-3}	0.622
Aromatic quality	m/z 581.1178	-	3.88×10^{-3}	0.602
Aromatic quality	m/z 668.2197	-	6.27×10^{-3}	0.576
Aromatic quality	Pelargonidin 3-(6''-succinyl-glucoside)	Flavonoids	6.27×10^{-3}	0.576
Aromatic quality	sinapaldehyde glucoside	Carbohydrates	9.68×10^{-3}	0.551
Aromatic quality	m/z 713.2618	-	1.12×10^{-2}	0.542
Aromatic quality	m/z 884.9105	-	1.33×10^{-2}	0.531
Aromatic quality	Inosine	Carbohydrates	1.35×10^{-2}	0.53
Aromatic quality	m/z 850.5936	-	1.76×10^{-2}	0.512
Aromatic quality	UDP-D-galacturonate	Carbohydrates	1.88×10^{-2}	0.508

Aromatic quality	DGTS(15:3/22:7)	Lipids [GP]	8.97×10^{-4}	-0.67
Aromatic quality	PS(17:0/18:3(9Z,12Z,15Z))	Lipids [GP]	1.81×10^{-3}	-0.639
Aromatic quality	m/z 735.3842	-	1.84×10^{-3}	-0.638
Aromatic quality	Sinapaldehyde glucoside	Carbohydrates	2.89×10^{-3}	-0.617
Aromatic quality	m/z 850.7066	-	3.50×10^{-3}	-0.607
Aromatic quality	m/z 727.9535	-	3.78×10^{-3}	-0.603
Aromatic quality	m/z 792.6708	-	3.78×10^{-3}	-0.603
Aromatic quality	3-Galloylprocyanidin B1	Flavonoids	4.18×10^{-3}	-0.598
Aromatic quality	Trigonelline	Alkaloids	4.55×10^{-3}	-0.594
Aromatic quality	m/z 732.7101	-	4.90×10^{-3}	-0.59
Aromatic quality	m/z 674.8121	-	5.20×10^{-3}	-0.586
Aromatic quality	Quercetin 4´-glucoside	Flavonoids	5.33×10^{-3}	-0.585
Aromatic quality	DGTS(16:0/26:5)	Lipids [GP]	5.78×10^{-3}	-0.581
Aromatic quality	m/z 855.8055	-	6.06×10^{-3}	-0.578
Aromatic quality	N-Acetylglucosamine 6-phosphate	Carbohydrates	6.06×10^{-3}	-0.578
Aromatic quality	TG(16:0/16:1/16:1)	Lipids [GL]	6.72×10^{-3}	-0.572
Aromatic quality	Astaxanthin diglucoside	Lipids [PR]	7.61×10^{-3}	-0.565
Aromatic quality	PS(20:2(11Z,14Z)/22:1(11Z))	Lipids [GP]	7.96×10^{-3}	-0.562
Aromatic quality	7-deoxyloganin	Lipids [PR]	8.04×10^{-3}	-0.562
Aromatic quality	m/z 876.5869	-	9.17×10^{-3}	-0.554
Aromatic quality	m/z 688.7849	-	1.11×10^{-2}	-0.542
Aromatic quality	m/z 520.6461	-	1.17×10^{-2}	-0.539
Aromatic quality	m/z 864.6799	-	1.17×10^{-2}	-0.539
Aromatic quality	PA(O-20:0/20:3)	Lipids [GP]	1.23×10^{-2}	-0.536
Aromatic quality	m/z 994.4843	-	1.28×10^{-2}	-0.533
Aromatic quality	m/z 743.4957	-	1.31×10^{-2}	-0.532
Aromatic quality	UDP-D-galactose	Carbohydrates	1.45×10^{-2}	-0.525
Aromatic quality	3-Feruloylquinic acid	Chlorogenic acids	1.56×10^{-2}	-0.52
Aromatic quality	geranylfarnesyl diphosphate	Lipids [PR]	1.67×10^{-2}	-0.516
Aromatic quality	m/z 963.4788	-	1.66×10^{-2}	-0.516
Aromatic quality	PC(16:0/16:0)	Lipids [GP]	1.81×10^{-2}	-0.51
Aromatic quality	m/z 756.6541	-	2.02×10^{-2}	-0.503
Aromatic quality	m/z 742.3064	-	2.04×10^{-2}	-0.502
Aromatic quality	PS(22:0/18:3(6Z,9Z,12Z))	Lipids [GP]	2.06×10^{-2}	-0.501
Aromatic quality	DGTS(11:1/16:5)	Lipids [GL]	2.10×10^{-2}	-0.5
Bitterness	Glutaryl-CoA	Fatty acids	1.63×10^{-4}	0.732
Bitterness	LacCer(d18:0/22:0)	Lipids [SP]	1.91×10^{-4}	0.727
Bitterness	PS(20:2(11Z,14Z)/22:1(11Z))	Lipids [GP]	2.41×10^{-4}	0.719
Bitterness	m/z 854.7416	-	3.90×10^{-4}	0.702
Bitterness	m/z 850.5936	-	4.49×10^{-4}	0.697
Bitterness	Undecaprenyl diphosphate	Lipids [PR]	4.57×10^{-4}	0.696
Bitterness	m/z 886.9232	-	8.33×10^{-4}	0.673
Bitterness	Apigenin 6-C-arabinoside 8-C-glucoside	Flavonoids	1.32×10^{-3}	0.653
Bitterness	m/z 665.7307	-	1.49×10^{-3}	0.648
Bitterness	All-trans-dodecaprenyl diphosphate	Lipids [PR]	1.51×10^{-3}	0.647
Bitterness	Pelargonidin 3-(6''-succinyl-glucoside)	Flavonoids	1.72×10^{-3}	0.642
Bitterness	m/z 821.605	-	1.87×10^{-3}	0.638

Bitterness	m/z 693.5954	-	2.40×10^{-3}	0.626
Bitterness	Man3GlcNAcFucGlcNAc	Carbohydrates	2.50×10^{-3}	0.624
Bitterness	Undecaprenyl diphosphate	Lipids [PR]	3.70×10^{-3}	0.604
Bitterness	m/z 552.6861	-	4.63×10^{-3}	0.593
Bitterness	m/z 884.9105	-	5.03×10^{-3}	0.588
Bitterness	m/z 668.2197	-	5.16×10^{-3}	0.587
Bitterness	m/z 713.2618	-	5.28×10^{-3}	0.586
Bitterness	m/z 538.0293	-	5.74×10^{-3}	0.581
Bitterness	Quercetin -(2''-(E)-caffeoyl-alpha-L-arabinopyranosyl) -(1->6)-glucoside	Flavonoids	6.44×10^{-3}	0.575
Bitterness	m/z 733.0316	-	6.74×10^{-3}	0.572
Bitterness	m/z 805.6282	-	7.14×10^{-3}	0.569
Bitterness	TG(18:2/18:3/20:0)[iso6]	Lipids [GL]	9.20×10^{-3}	0.554
Bitterness	a-Zeacarotene	Carotenoids	9.71×10^{-3}	0.551
Bitterness	Naphthalene-1,2-diol	Hydrocarbons	1.17×10^{-2}	0.539
Bitterness	m/z 740.3981	-	1.30×10^{-2}	0.532
Bitterness	m/z 743.4957	-	1.32×10^{-2}	0.531
Bitterness	UDP-3-O-(3-hydroxymyristoyl)-N-acetylglucosamine)	Carbohydrates	1.34×10^{-2}	0.53
Bitterness	m/z 667.0015	-	1.90×10^{-2}	0.507
Bitterness	PS(12:0/15:0)	Lipids [GP]	2.05×10^{-2}	0.502
Bitterness	m/z 727.9535	-	1.07×10^{-4}	-0.745
Bitterness	m/z 963.4788	-	2.55×10^{-4}	-0.717
Bitterness	m/z 498.7039	-	1.14×10^{-3}	-0.66
Bitterness	Quercetin 4′-glucoside	Flavonoids	3.52×10^{-3}	-0.607
Bitterness	3-keto-4-methylzymosterol	Lipids [ST]	4.63×10^{-3}	-0.593
Bitterness	Trigonelline	Alkaloids	5.60×10^{-3}	-0.582
Bitterness	m/z 722.7304	-	5.74×10^{-3}	-0.581
Bitterness	m/z 966.3254	-	6.82×10^{-3}	-0.571
Bitterness	Astaxanthin diglucoside	Lipids [PR]	7.98×10^{-3}	-0.562
Bitterness	PC(18:3(6Z,9Z,12Z)/18:1(9Z))	Lipids [GP]	1.10×10^{-2}	-0.543
Bitterness	m/z 822.7914	-	1.15×10^{-2}	-0.54
Bitterness	m/z 679.6623	-	1.27×10^{-2}	-0.534
Body	m/z 713.2618	-	1.24×10^{-4}	0.74
Body	m/z 886.9232	-	6.44×10^{-4}	0.683
Body	Man3GlcNAcFucGlcNAc	Carbohydrates	5.73×10^{-3}	0.581
Body	m/z 854.7416	-	6.74×10^{-3}	0.572
Body	m/z 581.1178	-	9.21×10^{-3}	0.554
Body	m/z 690.8389	-	1.13×10^{-2}	0.541
Body	Calcium (+)-pantothenate	Organic acids	1.25×10^{-2}	0.535
Body	m/z 884.9105	-	1.26×10^{-2}	0.534
Body	TG(14:0/20:5/20:5)	Lipids [GL]	1.32×10^{-2}	0.532
Body	m/z 665.7307	-	1.32×10^{-2}	0.532
Body	m/z 857.0291	-	1.45×10^{-2}	0.525
Body	m/z 914.9457	-	1.77×10^{-2}	0.512
Body	PE(18:4(6Z,9Z,12Z,15Z)/P-16:0)	Lipids [GP]	1.91×10^{-2}	0.507
Body	Trigonelline	Alkaloids	7.00×10^{-5}	-0.757
Body	5,9-tetracosadienoic acid	Fatty acids	6.55×10^{-4}	-0.682
Body	3-Caffeoyl-4-sinapoylquinic acid	Chlorogenic acids	9.35×10^{-4}	-0.668

Body	PC(18:3(6Z,9Z,12Z)/18:1(9Z))	Lipids [GP]	1.04×10^{-3}	-0.663
Body	Pelargonidin-3,5-diglucoside	Flavonoids	1.16×10^{-3}	-0.659
Body	PA(12:0/12:0)	Lipids [GP]	1.25×10^{-3}	-0.656
Body	Neoxanthin	Carotenoids	1.64×10^{-3}	-0.644
Body	Quercetin 4´-glucoside	Flavonoids	1.89×10^{-3}	-0.637
Body	m/z 522.9324	-	1.97×10^{-3}	-0.635
Body	DGTS(16:0/26:5)	Lipids [GP]	2.20×10^{-3}	-0.63
Body	Lycopene	Carotenoids	2.66×10^{-3}	-0.621
Body	m/z 868.6639	-	2.70×10^{-3}	-0.62
Body	m/z 822.7996	-	2.85×10^{-3}	-0.618
Body	Citric acid	Organic acids	2.96×10^{-3}	-0.616
Body	m/z 818.6563	-	3.25×10^{-3}	-0.611
Body	PE(22:2/24:1)	Lipids [GP]	3.37×10^{-3}	-0.609
Body	m/z 954.7218	-	3.37×10^{-3}	-0.609
Body	m/z 544.8249	-	4.34×10^{-3}	-0.596
Body	PC(18:2(2E,4E)/0:0)	Lipids [GP]	5.21×10^{-3}	-0.586
Body	PC(16:0/18:1(9Z))	Lipids [GP]	5.34×10^{-3}	-0.585
Body	m/z 986.6928	-	5.40×10^{-3}	-0.584
Body	DGTS(10:0/10:1)	Lipids [GL]	5.53×10^{-3}	-0.583
Body	DGTS(10:2/15:3)	Lipids [GL]	5.87×10^{-3}	-0.58
Body	m/z 706.7726	-	6.29×10^{-3}	-0.576
Body	PC(18:1(11Z)/18:1(9Z))	Lipids [GP]	6.37×10^{-3}	-0.575
Body	TG(20:2(11Z,14Z)	Lipids [GL]	6.51×10^{-3}	-0.574
Body	m/z 576.0403	-	6.98×10^{-3}	-0.57
Body	m/z 498.8341	-	7.38×10^{-3}	-0.567
Body	m/z 924.7318	-	7.38×10^{-3}	-0.567
Body	m/z 690.8471	-	7.55×10^{-3}	-0.565
Body	m/z 618.8237	-	7.72×10^{-3}	-0.564
Body	m/z 736.7362	-	7.90×10^{-3}	-0.563
Body	m/z 743.6496	-	8.26×10^{-3}	-0.56
Body	TG 51:0; TG(16:0/16:0/19:0)	Lipids [GL]	9.82×10^{-3}	-0.55
Body	Cer(d14:2(4E,6E)/22:1(13Z)(2OH))	Lipids [SP]	1.00×10^{-2}	-0.548
Body	Caffeoylcholine	Esters	1.03 x 10-2	-0.547
Body	m/z 437.2850	-	1.07×10^{-2}	-0.545
Body	m/z 750.7424	-	1.10×10^{-2}	-0.543
Body	1-Palmitoylglycerophosphocholine	Lipids [GP]	1.12×10^{-2}	-0.542
Body	m/z 778.8174	-	1.17×10^{-2}	-0.539
Body	m/z 971.5906	-	1.20×10^{-2}	-0.537
Body	Isoarachidic acid	Fatty acids	1.24×10^{-2}	-0.535
Body	UDP-D-galactose	Carbohydrates	1.36×10^{-2}	-0.53
Body	TG(16:1/18:1/18:1)	Lipids [GL]	1.40×10^{-2}	-0.528
Body	PC(24:0/P-18:1(11Z))	Lipids [GP]	1.53×10^{-2}	-0.522
Body	PC(18:0/18:2(9Z,12Z))	Lipids [GP]	1.56×10^{-2}	-0.52
Body	Astaxanthin diglucoside	Lipids [PR]	1.60×10^{-2}	-0.518
Body	Cer(d18:0/26:0)	Lipids [SP]	1.75×10^{-2}	-0.513
Body	m/z 826.3389	-	1.75×10^{-2}	-0.513
Body	m/z 997.5751	-	1.75×10^{-2}	-0.513

Body	TG(18:2/18:2/18:2)	Lipids [GL]	2.02 x 10^{-2}	-0.503
Body	m/z 794.7284	-	2.00 x 10^{-2}	-0.503
Flavor	Pelargonidin 3-(6''-succinyl-glucoside)	Flavonoids	2.68 x 10^{-4}	0.715
Flavor	m/z 665.7307	-	8.03 x 10^{-4}	0.674
Flavor	m/z 854.7416	-	2.77 x 10^{-3}	0.619
Flavor	UDP-D-xylose	Carbohydrates	3.29 x 10^{-3}	0.611
Flavor	m/z 668.2197	-	3.60 x 10^{-3}	0.606
Flavor	m/z 713.2618	-	4.08 x 10^{-3}	0.599
Flavor	Glutaryl-CoA	Fatty acids	6.80 x 10^{-3}	0.572
Flavor	m/z 992.6853	-	7.28 x 10^{-3}	0.568
Flavor	m/z 737.1965	-	7.61 x 10^{-3}	0.565
Flavor	m/z 886.9232	-	7.61 x 10^{-3}	0.565
Flavor	m/z 850.5936	-	1.04 x 10^{-2}	0.546
Flavor	Hederagenin	Terpenoids	1.28 x 10^{-2}	0.533
Flavor	m/z 884.9105	-	1.64 x 10^{-2}	0.517
Flavor	m/z 727.9535	-	1.71 x 10^{-3}	-0.642
Flavor	DGTS(16:0/26:5)	Lipids [GP]	2.24 x 10^{-3}	-0.629
Flavor	Quercetin 4´-glucoside	Flavonoids	7.53 x 10^{-3}	-0.566
Flavor	Astaxanthin diglucoside	Lipids [PR]	9.47 x 10^{-3}	-0.552
Flavor	m/z 722.7304	-	9.99 x 10^{-3}	-0.549
Flavor	m/z 963.4788	-	1.43 x 10^{-2}	-0.526
Flavor	Trigonelline	Alkaloids	1.47 x 10^{-2}	-0.524
Flavor	m/z 846.6485	-	1.82 x 10^{-2}	-0.51
Global preference	m/z 886.9232	-	6.29 x 10^{-5}	0.76
Global preference	Pelargonidin 3-(6''-succinyl-glucoside)	Flavonoids	1.17 x 10^{-4}	0.742
Global preference	m/z 665.7307	-	2.80 x 10^{-4}	0.714
Global preference	Glutaryl-CoA	Fatty acids	4.41 x 10^{-4}	0.697
Global preference	m/z 713.2618	-	4.41 x 10^{-4}	0.697
Global preference	m/z 854.7416	-	8.88 x 10^{-4}	0.67
Global preference	m/z 884.9105	-	9.76 x 10^{-4}	0.666
Global preference	Man3GlcNAcFucGlcNAc	Carbohydrates	2.21 x 10^{-3}	0.63
Global preference	Undecaprenyl diphosphate	Lipids [PR]	2.68 x 10^{-3}	0.621
Global preference	Apigenin 6-C-arabinoside 8-C-glucoside	Flavonoids	3.17 x 10^{-3}	0.612
Global preference	UDP-D-xylose	Carbohydrates	3.17 x 10^{-3}	0.612
Global preference	m/z 668.2197	-	3.80 x 10^{-3}	0.603
Global preference	Quercetin -(2''-(E)-caffeoyl-alpha-L-arabinopyranosyl) -(1->6)-glucoside	Flavonoids	6.08 x 10^{-3}	0.578
Global preference	m/z 737.1965	-	6.52 x 10^{-3}	0.574
Global preference	PS(20:2(11Z,14Z)/22:1(11Z))	Lipids [GP]	7.47 x 10^{-3}	0.566
Global preference	m/z 690.8389	-	7.81 x 10^{-3}	0.564
Global preference	All-trans-dodecaprenyl diphosphate	Lipids [PR]	7.89 x 10^{-3}	0.563
Global preference	Undecaprenyl diphosphate	Lipids [PR]	9.00 x 10^{-3}	0.555
Global preference	Phytofluene	Carotenoids	1.15 x 10^{-2}	0.54
Global preference	m/z 693.5954	-	1.29 x 10^{-2}	0.533
Global preference	Hederagenin	Terpenoids	1.37 x 10^{-2}	0.529
Global preference	a-Zeacarotene	Carotenoids	1.57 x 10^{-2}	0.52
Global preference	m/z 850.5936	-	1.60 x 10^{-2}	0.519
Global preference	m/z 805.6282	-	1.83 x 10^{-2}	0.51

Global preference	LacCer(d18:0/22:0)	Lipids [SP]	1.94 x 10^{-2}	0.506
Global preference	m/z 733.5719	-	2.07 x 10^{-2}	0.501
Global preference	m/z 727.9535	-	3.38 x 10^{-5}	-0.777
Global preference	Astaxanthin diglucoside	Lipids [PR]	3.50 x 10^{-4}	-0.706
Global preference	Trigonelline	Alkaloids	7.32 x 10^{-4}	-0.678
Global preference	m/z 963.4788	-	9.91 x 10^{-4}	-0.666
Global preference	Quercetin 4′-glucoside	Flavonoids	1.93 x 10^{-3}	-0.636
Global preference	m/z 729.2108	-	2.21 x 10^{-3}	-0.63
Global preference	PC(18:3(6Z,9Z,12Z)/18:1(9Z))	Lipids [GP]	2.94 x 10^{-3}	-0.616
Global preference	DGTS(16:0/26:5)	Lipids [GP]	3.99 x 10^{-3}	-0.601
Global preference	Neoxanthin	Carotenoids	4.57 x 10^{-3}	-0.593
Global preference	Pelargonidin-3,5-diglucoside	Flavonoids	4.68 x 10^{-3}	-0.592
Global preference	PA(12:0/12:0)	Lipids [GP]	8.07 x 10^{-3}	-0.562
Global preference	3-keto-4-methylzymosterol	Lipids [ST]	9.20 x 10^{-3}	-0.554
Global preference	3-Galloylprocyanidin B1	Flavonoids	1.00 x 10^{-2}	-0.549
Global preference	m/z 846.6485	-	1.27 x 10^{-2}	-0.534
Global preference	5,9-tetracosadienoic acid	Fatty acids	1.35 x 10^{-2}	-0.53
Global preference	m/z 743.6496	-	1.49 x 10^{-2}	-0.523
Global preference	m/z 498.7039	-	1.52 x 10^{-2}	-0.522
Global preference	m/z 722.7304	-	1.52 x 10^{-2}	-0.522
Global preference	m/z 468.5869	-	1.94 x 10^{-2}	-0.506
Sourness	m/z 727.9535	-	3.05 x 10^{-4}	0.711
Sourness	m/z 729.2108	-	1.19 x 10^{-3}	0.658
Sourness	Quercetin 4′-glucoside	Flavonoids	1.67 x 10^{-3}	0.643
Sourness	Astaxanthin diglucoside	Lipids [PR]	2.89 x 10^{-3}	0.617
Sourness	m/z 963.4788	-	3.13 x 10^{-3}	0.613
Sourness	m/z 498.7039	-	4.62 x 10^{-3}	0.593
Sourness	m/z 722.7304	-	6.08 x 10^{-3}	0.578
Sourness	3-keto-4-methylzymosterol	Lipids [ST]	8.72 x 10^{-3}	0.557
Sourness	PG(18:0/18:3(6Z,9Z,12Z))	Lipids [GP]	1.51 x 10^{-2}	0.522
Sourness	Tricetin 7,3',4',5'-trimethyl eter 5-xylosyl-(1->2)-rhamnoside	Flavonoids	1.67 x 10^{-2}	0.516
Sourness	PC(18:3(6Z,9Z,12Z)/18:1(9Z))	Lipids [GP]	1.96 x 10^{-2}	0.505
Sourness	Glutaryl-CoA	Fatty acids	6.64 x 10^{-5}	-0.759
Sourness	m/z 886.9232	-	1.96 x 10^{-4}	-0.726
Sourness	Apigenin 6-C-arabinoside 8-C-glucoside	Flavonoids	2.94 x 10^{-4}	-0.712
Sourness	Pelargonidin 3-(6''-succinyl-glucoside)	Flavonoids	3.28 x 10^{-4}	-0.708
Sourness	Undecaprenyl diphosphate	Lipids [PR]	5.87 x 10^{-4}	-0.687
Sourness	m/z 632.5865	-	5.96 x 10^{-4}	-0.686
Sourness	a-Zeacarotene	Carotenoids	9.56 x 10^{-4}	-0.667
Sourness	Man3GlcNAcFucGlcNAc	Carbohydrates	1.17 x 10^{-3}	-0.659
Sourness	m/z 854.7416	-	1.53 x 10^{-3}	-0.647
Sourness	m/z 821.605	-	1.87 x 10^{-3}	-0.638
Sourness	m/z 884.9105	-	1.95 x 10^{-3}	-0.636
Sourness	m/z 850.5936	-	2.30 x 10^{-3}	-0.628
Sourness	m/z 668.2197	-	2.50 x 10^{-3}	-0.624
Sourness	m/z 665.7307	-	3.05 x 10^{-3}	-0.614
Sourness	Quercetin -(2''-(E)-caffeoyl-alpha-L-arabinopyranosyl) -(1->6)-glucoside	Flavonoids	3.25 x 10^{-3}	-0.611

Sourness	m/z 690.8389	-	3.38×10^{-3}	-0.609
Sourness	PS(20:2(11Z,14Z)/22:1(11Z))	Lipids [GP]	3.89×10^{-3}	-0.602
Sourness	m/z 713.2618	-	4.57×10^{-3}	-0.593
Sourness	m/z 733.0316	-	4.74×10^{-3}	-0.592
Sourness	Undecaprenyl diphosphate	Lipids [PR]	4.97×10^{-3}	-0.589
Sourness	m/z 896.5138	-	5.22×10^{-3}	-0.586
Sourness	Phytofluene	Carotenoids	6.01×10^{-3}	-0.579
Sourness	m/z 667.0015	-	6.67×10^{-3}	-0.573
Sourness	PG(17:2(9Z,12Z)/17:2(9Z,12Z))	Lipids [GP]	6.90×10^{-3}	-0.571
Sourness	LacCer(d18:0/22:0)	Lipids [SP]	6.98×10^{-3}	-0.57
Sourness	All-trans-dodecaprenyl diphosphate	Lipids [PR]	7.06×10^{-3}	-0.569
Sourness	UDP-3-O-(3-hydroxymyristoyl)-N-acetylglucosamine)	Carbohydrates	1.00×10^{-2}	-0.549
Sourness	UDP-D-xylose	Carbohydrates	1.33×10^{-2}	-0.531
Sourness	m/z 805.6282	-	1.35×10^{-2}	-0.53
Sourness	m/z 866.5993	-	1.45×10^{-2}	-0.525
Sourness	TG(18:2/18:3/20:0)[iso6]	Lipids [GL]	1.47×10^{-2}	-0.524
Sourness	m/z 693.5954	-	1.50×10^{-2}	-0.523
Sourness	m/z 802.7472	-	1.80×10^{-2}	-0.511
Sourness	acetylmuramyl-alanyl-isoglutamine	Carbohydrates	1.98×10^{-2}	-0.504

Sensory attributes are presented in alphabetic order and metabolites are shown in decreasing order of Spearman Correlation Coefficient (SCC) for both positive and negative correlation.

Table S2. Highly heritable metabolites of coffee fruits and coffee leaf linked to sensory traits of coffee beverage.

Sensory attribute	*m/z*	Ionization mode	Tissue	Heritability ± SE	SCC	*p*-Value
Acidity	791.2383	+	Fruits	0.984 ± 0.042	-0.750	1.99×10^{-2}
Acidity	963.4134	+	Fruits	0.960 ± 0.027	0.667	4.99×10^{-2}
Acidity	597.605	-	Fruits	0.839 ± 0.165	-0.767	1.59×10^{-2}
Acidity	559.3646	-	Fruits	0.741 ± 0.085	0.700	3.58×10^{-2}
Acidity	678.3575	+	Fruits	0.605 ± 0.089	-0.733	2.46×10^{-2}
Acidity	635.6373	-	Fruits	0.575 ± 0.117	0.700	3.58×10^{-2}
Acidity	879.5471	-	Fruits	0.551 ± 0.174	-0.667	4.99×10^{-2}
Acidity	611.862	+	Fruits	0.509 ± 0.124	0.750	1.99×10^{-2}
Acidity	140.0413	+	Fruits	0.464 ± 0.098	-0.817	7.22×10^{-3}
Acidity	376.4213	-	Fruits	0.458 ± 0.125	-0.733	2.46×10^{-2}
Acidity	528.7035	+	Fruits	0.432 ± 0.170	-0.867	2.50×10^{-3}
Acidity	513.9542	+	Fruits	0.402 ± 0.170	0.683	4.24×10^{-2}
Aftertaste	940.6771	+	Fruits	0.881 ± 0.075	0.754	1.88×10^{-2}
Aftertaste	573.676	+	Fruits	0.797 ± 0.141	-0.746	2.10×10^{-2}

Aftertaste	609.9023	+	Fruits	0.765 ± 0.064	0.780	1.32 x 10^{-2}
Aftertaste	623.5736	+	Fruits	0.762 ± 0.090	-0.678	4.47 x 10^{-2}
Aftertaste	559.3646	-	Fruits	0.741 ± 0.085	-0.712	3.14 x 10^{-2}
Aftertaste	883.1307	-	Fruits	0.689 ± 0.108	-0.712	3.14 x 10^{-2}
Aftertaste	597.4666	+	Fruits	0.674 ± 0.095	-0.822	6.53 x 10^{-3}
Aftertaste	875.5625	-	Fruits	0.674 ± 0.164	-0.678	4.47 x 10^{-2}
Aftertaste	536.0019	+	Fruits	0.656 ± 0.098	0.695	3.77 x 10^{-2}
Aftertaste	353.8873	+	Fruits	0.655 ± 0.261	0.737	2.34 x 10^{-2}
Aftertaste	500.5095	+	Fruits	0.652 ± 0.096	-0.670	4.85 x 10^{-2}
Aftertaste	597.6393	-	Fruits	0.615 ± 0.098	0.780	1.32 x 10^{-2}
Aftertaste	166.4223	+	Fruits	0.614 ± 0.114	-0.729	2.59 x 10^{-2}
Aftertaste	611.064	+	Fruits	0.515 ± 0.068	-0.695	3.77 x 10^{-2}
Aftertaste	939.369	-	Fruits	0.481 ± 0.180	0.788	1.16 x 10^{-2}
Aftertaste	367.7395	+	Fruits	0.446 ± 0.115	0.890	1.30 x 10^{-3}
Aromatic intensity	860.5079	-	Fruits	0.999 ± 0.229	0.812	7.89 x 10^{-3}
Aromatic intensity	532.8961	+	Fruits	0.986 ± 0.101	0.678	4.48 x 10^{-2}
Aromatic intensity	595.6598	-	Fruits	0.986 ± 0.140	0.703	3.47 x 10^{-2}
Aromatic intensity	571.6767	-	Fruits	0.983 ± 0.161	0.703	3.47 x 10^{-2}
Aromatic intensity	839.0698	+	Fruits	0.902 ± 0.090	-0.711	3.17 x 10^{-2}
Aromatic intensity	512.7222	+	Fruits	0.783 ± 0.205	-0.828	5.80 x 10^{-3}
Aromatic intensity	665.8433	+	Fruits	0.776 ± 0.093	0.703	3.47 x 10^{-2}
Aromatic intensity	837.855	+	Fruits	0.775 ± 0.073	-0.695	3.79 x 10^{-2}
Aromatic intensity	724.9043	+	Fruits	0.771 ± 0.183	-0.929	2.90 x 10^{-4}
Aromatic intensity	637.0151	+	Fruits	0.768 ± 0.102	0.703	3.47 x 10^{-2}
Aromatic intensity	687.6503	+	Fruits	0.754 ± 0.214	-0.669	4.86 x 10^{-2}
Aromatic intensity	715.847	+	Fruits	0.734 ± 0.067	-0.703	3.47 x 10^{-2}
Aromatic intensity	372.7583	+	Fruits	0.712 ± 0.077	0.678	4.48 x 10^{-2}
Aromatic intensity	624.8226	+	Fruits	0.701 ± 0.089	0.686	4.12 x 10^{-2}
Aromatic intensity	103.135	+	Fruits	0.652 ± 0.097	-0.736	2.37 x 10^{-2}
Aromatic intensity	712.0247	+	Fruits	0.652 ± 0.082	-0.862	2.81 x 10^{-3}
Aromatic intensity	710.9163	+	Fruits	0.631 ± 0.094	-0.879	1.82 x 10^{-3}
Aromatic intensity	705.6388	-	Fruits	0.630 ± 0.068	0.686	4.12 x 10^{-2}
Aromatic intensity	486.3118	+	Fruits	0.612 ± 0.089	-0.686	4.12 x 10^{-2}
Aromatic intensity	696.3253	-	Fruits	0.566 ± 0.125	-0.695	3.79 x 10^{-2}
Aromatic intensity	513.746	-	Fruits	0.537 ± 0.086	0.812	7.89 x 10^{-3}
Aromatic intensity	856.5722	-	Fruits	0.514 ± 0.144	0.803	9.11 x 10^{-3}
Aromatic intensity	663.8415	+	Fruits	0.503 ± 0.145	0.686	4.12 x 10^{-2}
Aromatic intensity	861.7196	-	Fruits	0.502 ± 0.105	0.678	4.48 x 10^{-2}
Aromatic intensity	805.8779	-	Fruits	0.488 ± 0.081	0.695	3.79 x 10^{-2}
Aromatic intensity	577.8055	+	Fruits	0.470 ± 0.085	0.879	1.82 x 10^{-3}
Aromatic intensity	563.2989	+	Fruits	0.463 ± 0.090	0.946	1.20 x 10^{-4}
Aromatic intensity	492.4978	+	Fruits	0.440 ± 0.079	-0.695	3.79 x 10^{-2}
Aromatic intensity	762.7093	+	Fruits	0.409 ± 0.160	-0.703	3.47 x 10^{-2}
Aromatic quality	794.6727	+	Fruits	0.998 ± 0.094	-0.717	2.98 x 10^{-2}
Aromatic quality	757.8108	+	Fruits	0.990 ± 0.142	-0.783	1.25 x 10^{-2}
Aromatic quality	757.7016	+	Fruits	0.966 ± 0.158	-0.700	3.58 x 10^{-2}
Aromatic quality	963.4134	+	Fruits	0.960 ± 0.027	-0.667	4.99 x 10^{-2}

Aromatic quality	788.6958	+	Fruits	0.949 ± 0.269	-0.867	2.50×10^{-3}
Aromatic quality	761.6315	+	Fruits	0.876 ± 0.173	-0.850	3.70×10^{-3}
Aromatic quality	885.1048	-	Fruits	0.845 ± 0.096	-0.683	4.24×10^{-2}
Aromatic quality	521.5618	+	Fruits	0.842 ± 0.108	-0.767	1.59×10^{-2}
Aromatic quality	729.6311	-	Fruits	0.836 ± 0.104	-0.833	5.27×10^{-3}
Aromatic quality	780.6749	+	Fruits	0.808 ± 0.154	-0.733	2.46×10^{-2}
Aromatic quality	512.7222	+	Fruits	0.783 ± 0.205	-0.817	7.22×10^{-3}
Aromatic quality	632.0464	+	Fruits	0.768 ± 0.229	-0.667	4.99×10^{-2}
Aromatic quality	885.4504	+	Fruits	0.764 ± 0.046	-0.750	1.99×10^{-2}
Aromatic quality	891.6844	-	Fruits	0.737 ± 0.127	-0.817	7.22×10^{-3}
Aromatic quality	691.972	+	Fruits	0.688 ± 0.107	-0.967	2.00 x 10-5
Aromatic quality	536.0019	+	Fruits	0.656 ± 0.098	0.800	9.63×10^{-3}
Aromatic quality	712.0247	+	Fruits	0.652 ± 0.082	-0.700	3.58×10^{-2}
Aromatic quality	805.6824	+	Fruits	0.645 ± 0.066	-0.733	2.46×10^{-2}
Aromatic quality	599.9172	+	Fruits	0.637 ± 0.056	0.717	2.98×10^{-2}
Aromatic quality	543.3742	-	Fruits	0.634 ± 0.137	0.700	3.58×10^{-2}
Aromatic quality	705.6388	-	Fruits	0.630 ± 0.068	0.750	1.99×10^{-2}
Aromatic quality	486.3118	+	Fruits	0.612 ± 0.089	-0.850	3.70×10^{-3}
Aromatic quality	407.6521	+	Fruits	0.604 ± 0.121	-0.817	7.22×10^{-3}
Aromatic quality	614.8476	+	Fruits	0.593 ± 0.102	0.717	2.98×10^{-2}
Aromatic quality	731.4041	-	Fruits	0.587 ± 0.121	0.783	1.25×10^{-2}
Aromatic quality	536.2469	+	Fruits	0.555 ± 0.113	-0.733	2.46×10^{-2}
Aromatic quality	620.2667	+	Fruits	0.552 ± 0.074	-0.800	9.63×10^{-3}
Aromatic quality	573.4349	-	Fruits	0.535 ± 0.130	0.733	2.46×10^{-2}
Aromatic quality	337.7305	-	Fruits	0.533 ± 0.109	0.867	2.50×10^{-3}
Aromatic quality	881.4225	-	Fruits	0.531 ± 0.069	0.817	7.22×10^{-3}
Aromatic quality	165.2623	+	Fruits	0.508 ± 0.129	-0.800	9.63×10^{-3}
Aromatic quality	350.5547	+	Fruits	0.508 ± 0.107	-0.683	4.24×10^{-2}
Aromatic quality	559.7048	+	Fruits	0.484 ± 0.143	-0.667	4.99×10^{-2}
Aromatic quality	531.2356	+	Fruits	0.429 ± 0.143	-0.683	4.24×10^{-2}
Aromatic quality	366.9359	+	Fruits	0.427 ± 0.126	0.800	9.63×10^{-3}
Aromatic quality	365.8207	-	Fruits	0.419 ± 0.133	0.700	3.58×10^{-2}
Aromatic quality	737.5399	-	Fruits	0.414 ± 0.117	0.717	2.98×10^{-2}
Aromatic quality	762.7093	+	Fruits	0.409 ± 0.160	-0.700	3.58×10^{-2}
Aromatic quality	793.4098	-	Fruits	0.408 ± 0.116	0.700	3.58×10^{-2}
Astringency	601.1261	+	Fruits	0.957 ± 0.218	0.833	5.27×10^{-3}
Astringency	469.435	+	Fruits	0.894 ± 0.108	0.733	2.46×10^{-2}
Astringency	609.9023	+	Fruits	0.765 ± 0.064	0.850	3.70×10^{-3}
Astringency	381.6571	+	Fruits	0.735 ± 0.115	-0.700	3.58×10^{-2}
Astringency	883.1307	-	Fruits	0.689 ± 0.108	-0.700	3.58×10^{-2}
Astringency	523.934	+	Fruits	0.686 ± 0.154	-0.667	4.99×10^{-2}
Astringency	319.9445	+	Fruits	0.634 ± 0.119	0.733	2.46×10^{-2}
Astringency	597.6393	-	Fruits	0.615 ± 0.098	0.783	1.25×10^{-2}
Astringency	599.9839	+	Fruits	0.612 ± 0.102	0.733	2.46×10^{-2}
Astringency	607.9679	+	Fruits	0.597 ± 0.098	0.783	1.25×10^{-2}
Astringency	919.7309	+	Fruits	0.584 ± 0.106	-0.700	3.58×10^{-2}
Astringency	516.7384	-	Fruits	0.581 ± 0.159	-0.817	7.22×10^{-3}

Astringency	669.4101	+	Fruits	0.567 ± 0.088	0.700	3.58×10^{-2}
Astringency	696.3253	-	Fruits	0.566 ± 0.125	-0.783	1.25×10^{-2}
Astringency	544.5006	-	Fruits	0.557 ± 0.171	-0.700	3.58×10^{-2}
Astringency	873.9837	-	Fruits	0.527 ± 0.086	0.717	2.98×10^{-2}
Astringency	856.5722	-	Fruits	0.514 ± 0.144	0.700	3.58×10^{-2}
Astringency	923.2353	-	Fruits	0.482 ± 0.332	-0.667	4.99×10^{-2}
Astringency	802.4506	+	Fruits	0.476 ± 0.077	0.783	1.25×10^{-2}
Astringency	683.6056	+	Fruits	0.476 ± 0.050	0.767	1.59×10^{-2}
Astringency	132.5465	+	Fruits	0.417 ± 0.070	0.883	1.59×10^{-3}
Bitterness	913.5948	+	Fruits	0.997 ± 0.208	-0.866	2.56×10^{-3}
Bitterness	679.9643	+	Fruits	0.993 ± 0.234	-0.672	4.73×10^{-2}
Bitterness	794.6727	+	Fruits	0.989 ± 0.094	-0.748	2.05×10^{-2}
Bitterness	882.7669	+	Fruits	0.971 ± 0.050	-0.756	1.84×10^{-2}
Bitterness	617.0208	+	Fruits	0.855 ± 0.093	-0.866	2.56×10^{-3}
Bitterness	521.5618	+	Fruits	0.842 ± 0.108	-0.706	3.36×10^{-2}
Bitterness	734.4907	-	Fruits	0.817 ± 0.098	0.740	2.28×10^{-2}
Bitterness	780.6749	+	Fruits	0.808 ± 0.154	-0.773	1.46×10^{-2}
Bitterness	872.2035	-	Fruits	0.792 ± 0.098	-0.882	1.63×10^{-3}
Bitterness	538.0196	+	Fruits	0.787 ± 0.147	-0.740	2.28×10^{-2}
Bitterness	764.8121	+	Fruits	0.781 ± 0.158	-0.672	4.73×10^{-2}
Bitterness	550.8873	+	Fruits	0.779 ± 0.080	-0.689	4.01×10^{-2}
Bitterness	750.7443	+	Fruits	0.754 ± 0.151	-0.672	4.73×10^{-2}
Bitterness	534.0056	+	Fruits	0.752 ± 0.135	-0.698	3.67×10^{-2}
Bitterness	559.3646	-	Fruits	0.741 ± 0.085	-0.748	2.05×10^{-2}
Bitterness	552.1076	+	Fruits	0.735 ± 0.083	-0.756	1.84×10^{-2}
Bitterness	681.7437	+	Fruits	0.682 ± 0.168	-0.840	4.56×10^{-3}
Bitterness	748.7588	+	Fruits	0.616 ± 0.176	-0.740	2.28×10^{-2}
Bitterness	327.4659	+	Fruits	0.606 ± 0.291	-0.773	1.46×10^{-2}
Bitterness	621.4599	+	Fruits	0.584 ± 0.078	-0.714	3.06×10^{-2}
Bitterness	391.4852	+	Fruits	0.584 ± 0.094	-0.740	2.28×10^{-2}
Bitterness	654.9806	+	Fruits	0.582 ± 0.174	-0.723	2.78×10^{-2}
Bitterness	500.0636	+	Fruits	0.559 ± 0.153	-0.790	1.13×10^{-2}
Bitterness	889.9878	-	Fruits	0.549 ± 0.090	0.773	1.46×10^{-2}
Bitterness	573.4349	-	Fruits	0.535 ± 0.130	0.689	4.01×10^{-2}
Bitterness	534.7273	+	Fruits	0.527 ± 0.098	-0.899	9.70×10^{-4}
Bitterness	165.2623	+	Fruits	0.508 ± 0.129	-0.773	1.46×10^{-2}
Bitterness	743.0557	+	Fruits	0.474 ± 0.118	-0.782	1.29×10^{-2}
Bitterness	513.9542	+	Fruits	0.402 ± 0.170	-0.849	3.81×10^{-3}
Body	598.5406	+	Fruits	0.998 ± 0.300	-0.724	2.76×10^{-2}
Body	788.6958	+	Fruits	0.949 ± 0.269	0.690	3.99×10^{-2}
Body	591.7859	+	Fruits	0.939 ± 0.117	0.783	1.26×10^{-2}
Body	574.1631	+	Fruits	0.932 ± 0.144	0.715	3.04×10^{-2}
Body	631.6777	+	Fruits	0.927 ± 0.081	0.690	3.99×10^{-2}
Body	653.9152	+	Fruits	0.873 ± 0.153	0.817	7.15×10^{-3}
Body	545.7043	-	Fruits	0.808 ± 0.194	-0.826	6.11×10^{-3}
Body	782.7895	+	Fruits	0.793 ± 0.228	0.690	3.99×10^{-2}
Body	891.6844	-	Fruits	0.737 ± 0.127	0.792	1.10×10^{-2}

Body	830.0813	+	Fruits	0.728 ± 0.090	0.826	6.11×10^{-3}
Body	817.073	+	Fruits	0.699 ± 0.070	-0.707	3.34×10^{-2}
Body	648.7114	+	Fruits	0.665 ± 0.068	0.775	1.42×10^{-2}
Body	543.3742	-	Fruits	0.634 ± 0.137	-0.732	2.49×10^{-2}
Body	723.0045	-	Fruits	0.611 ± 0.097	-0.834	5.17×10^{-3}
Body	590.0408	+	Fruits	0.588 ± 0.134	-0.809	8.32×10^{-3}
Body	645.0176	+	Fruits	0.546 ± 0.167	0.783	1.26×10^{-2}
Body	881.4225	-	Fruits	0.531 ± 0.069	-0.809	8.32×10^{-3}
Body	634.6612	+	Fruits	0.495 ± 0.073	-0.860	2.96×10^{-3}
Body	895.0076	+	Fruits	0.478 ± 0.045	-0.690	3.99×10^{-2}
Body	263.9915	+	Fruits	0.471 ± 0.106	0.894	1.16×10^{-3}
Body	676.0284	+	Fruits	0.461 ± 0.121	-0.690	3.99×10^{-2}
Body	275.05	+	Fruits	0.426 ± 0.154	0.690	3.99×10^{-2}
Body	365.8207	-	Fruits	0.419 ± 0.133	-0.672	4.72×10^{-2}
Body	611.6908	-	Fruits	0.418 ± 0.154	-0.715	3.04×10^{-2}
Flavor	598.5406	+	Fruits	0.998 ± 0.300	0.740	2.28×10^{-2}
Flavor	679.9643	+	Fruits	0.991 ± 0.234	-0.681	4.36×10^{-2}
Flavor	794.6727	+	Fruits	0.987 ± 0.094	-0.832	5.41×10^{-3}
Flavor	788.6958	+	Fruits	0.949 ± 0.269	-0.723	2.78×10^{-2}
Flavor	617.0208	+	Fruits	0.855 ± 0.093	-0.773	1.46×10^{-2}
Flavor	521.5618	+	Fruits	0.842 ± 0.108	-0.714	3.06×10^{-2}
Flavor	780.6749	+	Fruits	0.808 ± 0.154	-0.874	2.06×10^{-3}
Flavor	872.2035	-	Fruits	0.792 ± 0.098	-0.689	4.01×10^{-2}
Flavor	512.7222	+	Fruits	0.783 ± 0.205	-0.782	1.29×10^{-2}
Flavor	764.8121	+	Fruits	0.781 ± 0.158	-0.672	4.73×10^{-2}
Flavor	891.6844	-	Fruits	0.737 ± 0.127	-0.765	1.64×10^{-2}
Flavor	835.1677	-	Fruits	0.718 ± 0.120	-0.790	1.13×10^{-2}
Flavor	663.7809	+	Fruits	0.710 ± 0.110	-0.706	3.36×10^{-2}
Flavor	691.972	+	Fruits	0.688 ± 0.107	-0.756	1.84×10^{-2}
Flavor	536.0019	+	Fruits	0.656 ± 0.098	0.756	1.84×10^{-2}
Flavor	705.6388	-	Fruits	0.630 ± 0.068	0.731	2.52×10^{-2}
Flavor	805.5854	+	Fruits	0.621 ± 0.138	0.765	1.64×10^{-2}
Flavor	736.8592	+	Fruits	0.619 ± 0.237	-0.740	2.28×10^{-2}
Flavor	500.0636	+	Fruits	0.559 ± 0.153	-0.706	3.36×10^{-2}
Flavor	536.2469	+	Fruits	0.555 ± 0.113	-0.698	3.67×10^{-2}
Flavor	620.2667	+	Fruits	0.552 ± 0.074	-0.706	3.36×10^{-2}
Flavor	573.4349	-	Fruits	0.535 ± 0.130	0.706	3.36×10^{-2}
Flavor	337.7305	-	Fruits	0.533 ± 0.109	0.874	2.06×10^{-3}
Flavor	165.2623	+	Fruits	0.508 ± 0.129	-0.891	1.27×10^{-3}
Flavor	559.7048	+	Fruits	0.484 ± 0.143	-0.681	4.36×10^{-2}
Flavor	641.6338	-	Fruits	0.484 ± 0.106	-0.740	2.28×10^{-2}
Flavor	743.0557	+	Fruits	0.474 ± 0.118	-0.740	2.28×10^{-2}
Global preference	913.5948	+	Fruits	0.999 ± 0.208	-0.762	1.71×10^{-2}
Global preference	794.6727	+	Fruits	0.998 ± 0.094	-0.720	2.88×10^{-2}
Global preference	791.2383	+	Fruits	0.984 ± 0.042	0.728	2.62×10^{-2}
Global preference	999.461	+	Fruits	0.887 ± 0.048	-0.678	4.48×10^{-2}
Global preference	617.0208	+	Fruits	0.855 ± 0.093	-0.778	1.35×10^{-2}

Global preference	521.5618	+	Fruits	0.842 ± 0.108	-0.812	7.89 x 10^{-3}
Global preference	780.6749	+	Fruits	0.808 ± 0.154	-0.837	4.91 x 10^{-3}
Global preference	872.2035	-	Fruits	0.792 ± 0.098	-0.711	3.17 x 10^{-2}
Global preference	512.7222	+	Fruits	0.783 ± 0.205	-0.728	2.62 x 10^{-2}
Global preference	835.1677	-	Fruits	0.718 ± 0.120	-0.854	3.42 x 10^{-3}
Global preference	663.7809	+	Fruits	0.710 ± 0.110	-0.728	2.62 x 10^{-2}
Global preference	844.6292	+	Fruits	0.692 ± 0.154	0.711	3.17 x 10^{-2}
Global preference	391.4852	+	Fruits	0.584 ± 0.094	-0.686	4.12 x 10^{-2}
Global preference	337.7305	-	Fruits	0.533 ± 0.109	0.728	2.62 x 10^{-2}
Global preference	534.7273	+	Fruits	0.527 ± 0.098	-0.795	1.04 x 10^{-2}
Global preference	165.2623	+	Fruits	0.508 ± 0.129	-0.862	2.81 x 10^{-3}
Global preference	600.2095	+	Fruits	0.494 ± 0.126	-0.728	2.62 x 10^{-2}
Global preference	743.0557	+	Fruits	0.474 ± 0.118	-0.678	4.48 x 10^{-2}
Global preference	670.8873	+	Fruits	0.424 ± 0.090	-0.711	3.17 x 10^{-2}
Global preference	513.9542	+	Fruits	0.402 ± 0.170	-0.695	3.79 x 10^{-2}
Sourness	791.2383	+	Fruits	0.984 ± 0.042	-0.681	4.36 x 10^{-2}
Sourness	521.5618	+	Fruits	0.842 ± 0.108	0.689	4.01 x 10^{-2}
Sourness	512.7222	+	Fruits	0.783 ± 0.205	0.765	1.64 x 10^{-2}
Sourness	724.9043	+	Fruits	0.771 ± 0.183	0.740	2.28 x 10^{-2}
Sourness	835.1677	-	Fruits	0.718 ± 0.120	0.782	1.29 x 10^{-2}
Sourness	176.6859	+	Fruits	0.675 ± 0.062	0.765	1.64 x 10^{-2}
Sourness	138.5693	+	Fruits	0.553 ± 0.122	0.681	4.36 x 10^{-2}
Sourness	577.0395	+	Fruits	0.534 ± 0.151	0.765	1.64 x 10^{-2}
Sourness	534.7273	+	Fruits	0.527 ± 0.098	0.723	2.78 x 10^{-2}
Sourness	165.2623	+	Fruits	0.508 ± 0.129	0.756	1.84 x 10^{-2}
Sourness	670.8873	+	Fruits	0.424 ± 0.090	0.798	9.89 x 10^{-3}
Sourness	433.676	-	Fruits	0.401 ± 0.169	-0.807	8.60 x 10^{-3}
Acidity	433.1626	+	Leaf	0.996	-0.800	9.65 x 10^{-3}
Acidity	473.6946	+	Leaf	0.643	0.783	1.13 x 10^{-2}
Acidity	880.5206	+	Leaf	0.536	0.683	4.24 x 10^{-2}
Aftertaste	521.5735	+	Leaf	0.997	-0.711	3.14 x 10^{-2}
Aftertaste	898.6122	+	Leaf	0.992	-0.736	2.34 x 10^{-2}
Aftertaste	848.8565	+	Leaf	0.814	-0.771	1.49 x 10^{-2}
Aftertaste	901.7532	+	Leaf	0.686	-0.754	1.88 x 10^{-2}
Aftertaste	871.6361	+	Leaf	0.632	-0.889	1.30 x 10^{-3}
Aftertaste	937.54	+	Leaf	0.58	-0.881	1.68 x 10^{-3}
Aftertaste	905.8577	+	Leaf	0.561	-0.686	4.11 x 10^{-2}
Aftertaste	880.5206	+	Leaf	0.536	-0.823	6.49 x 10^{-3}
Aftertaste	695.3553	+	Leaf	0.535	0.737	2.41 x 10^{-2}
Aftertaste	953.8006	+	Leaf	0.508	-0.822	6.53 x 10^{-3}
Aftertaste	965.7206	+	Leaf	0.491	-0.737	2.47 x 10^{-2}
Aftertaste	589.8349	+	Leaf	0.471	-0.796	1.02 x 10^{-2}
Aftertaste	910.609	+	Leaf	0.417	-0.678	4.47 x 10^{-2}
Aromatic intensity	713.317	+	Leaf	0.958	0.803	9.01 x 10^{-3}
Aromatic intensity	717.7012	+	Leaf	0.832	0.744	2.13 x 10^{-2}
Aromatic intensity	195.6151	+	Leaf	0.682	0.761	1.71 x 10^{-2}
Aromatic intensity	387.3897	+	Leaf	0.539	0.677	4.48 x 10^{-2}

Aromatic quality	251.4295	+	Leaf	0.994	0.766	1.62×10^{-2}
Aromatic quality	713.317	+	Leaf	0.958	0.733	2.46×10^{-2}
Aromatic quality	517.7342	+	Leaf	0.909	-0.883	1.59×10^{-3}
Aromatic quality	463.8052	+	Leaf	0.891	0.683	4.24×10^{-2}
Aromatic quality	939.7826	+	Leaf	0.565	0.666	4.99×10^{-2}
Aromatic quality	933.5553	+	Leaf	0.564	0.781	1.23×10^{-2}
Aromatic quality	387.3897	+	Leaf	0.539	0.866	2.50×10^{-3}
Aromatic quality	695.3553	+	Leaf	0.535	0.816	7.22×10^{-3}
Aromatic quality	805.7835	+	Leaf	0.466	0.783	1.25×10^{-2}
Aromatic quality	545.5408	+	Leaf	0.414	0.758	1.59×10^{-2}
Astringency	885.6678	+	Leaf	0.999	-0.699	3.58×10^{-2}
Astringency	898.6122	+	Leaf	0.992	-0.683	4.24×10^{-2}
Astringency	931.6992	+	Leaf	0.901	-0.766	1.59×10^{-2}
Astringency	901.7532	+	Leaf	0.686	-0.916	5.10×10^{-4}
Astringency	852.961	+	Leaf	0.664	-0.663	4.99×10^{-2}
Astringency	871.6361	+	Leaf	0.632	-0.746	1.61×10^{-2}
Astringency	527.3898	+	Leaf	0.619	-0.800	9.63×10^{-3}
Astringency	967.7852	-	Leaf	0.612	0.666	4.90×10^{-2}
Astringency	905.8577	+	Leaf	0.561	-0.689	3.68×10^{-2}
Astringency	873.6858	+	Leaf	0.516	-0.801	9.53×10^{-3}
Bitterness	473.6946	+	Leaf	0.643	-0.857	3.14×10^{-3}
Bitterness	587.1469	+	Leaf	0.438	-0.941	1.50×10^{-4}
Body	463.8052	+	Leaf	0.891	-0.689	3.99×10^{-2}
Body	903.6637	+	Leaf	0.827	-0.752	2.02×10^{-2}
Body	794.4614	-	Leaf	0.783	-0.774	1.42×10^{-2}
Body	830.6143	+	Leaf	0.769	-0.927	3.10×10^{-4}
Body	863.1911	+	Leaf	0.584	-0.732	2.49×10^{-2}
Body	768.5363	+	Leaf	0.577	-0.681	4.34×10^{-2}
Body	939.7826	+	Leaf	0.565	-0.757	1.80×10^{-2}
Body	817.7302	+	Leaf	0.56	-0.749	2.32×10^{-2}
Body	805.7835	+	Leaf	0.466	-0.859	2.96×10^{-3}
Body	777.5542	+	Leaf	0.455	-0.791	1.10×10^{-2}
Body	929.8382	+	Leaf	0.419	-0.749	1.78×10^{-2}
Body	910.609	+	Leaf	0.417	-0.682	4.44×10^{-2}
Body	915.8301	+	Leaf	0.408	-0.749	2.12×10^{-2}
Flavor	251.4295	+	Leaf	0.994	0.672	4.89×10^{-2}
Flavor	517.7342	+	Leaf	0.909	-0.974	8.13×10^{-6}
Flavor	845.7379	+	Leaf	0.687	0.672	4.79×10^{-2}
Flavor	473.6946	+	Leaf	0.643	-0.705	3.36×10^{-2}
Flavor	387.3897	+	Leaf	0.539	0.669	4.73×10^{-2}
Flavor	695.3553	+	Leaf	0.535	0.798	9.89×10^{-3}
Flavor	823.431	+	Leaf	0.5	0.773	1.40×10^{-2}
Flavor	805.7835	+	Leaf	0.466	0.719	3.36×10^{-2}
Flavor	587.1469	+	Leaf	0.438	-0.747	2.05×10^{-2}
Global preference	433.1626	+	Leaf	0.996	0.677	4.48×10^{-2}
Global preference	251.4295	+	Leaf	0.994	0.702	3.47×10^{-2}
Global preference	517.7342	+	Leaf	0.909	-0.803	9.21×10^{-3}

Global preference	845.7379	+	Leaf	0.687	0.794	1.04×10^{-2}
Global preference	473.6946	+	Leaf	0.643	-0.920	4.30×10^{-4}
Global preference	387.3897	+	Leaf	0.539	0.711	3.17×10^{-2}
Global preference	695.3553	+	Leaf	0.535	0.778	1.35×10^{-2}
Global preference	587.1469	+	Leaf	0.438	-0.800	9.11×10^{-3}
Sourness	473.6946	+	Leaf	0.643	0.773	1.48×10^{-2}
Sourness	387.3897	+	Leaf	0.539	-0.705	3.36×10^{-2}
Sourness	695.3553	+	Leaf	0.535	-0.771	1.46×10^{-2}

Sensory attributes are presented by alphabetic order and metabolites are shown in decreasing order of heritability level for both positive and negative correlation. SE: Standard error; SCC: Spearman correlation coefficient.

9 by the authors. Submitted for possible open access publication under the terms and onditions of the Creative Commons Attribution (CC BY) license http://creativecommons.org/licenses/by/4.0/).
